# User-experiences with a web-based self-help intervention for partners of cancer patients based on acceptance and commitment therapy and self-compassion: a qualitative study

**DOI:** 10.1186/s12889-017-4121-2

**Published:** 2017-02-28

**Authors:** Nadine Köhle, Constance H. C. Drossaert, Jasmijn Jaran, Karlein M. G. Schreurs, Irma M. Verdonck-de Leeuw, Ernst T. Bohlmeijer

**Affiliations:** 1 Department of Psychology, Health & Technology, University of Twente, P.O. Box 217, 7500 AE Enschede, The Netherlands; 2 Roessingh Research & Development, P.O. Box 310, 7500 AH Enschede, The Netherlands; 3 Department of Clinical, Neuro-& Developmental Psychology, Section Clinical Psychology, VU University, van der Boechorststraat 1, 1082 BT Amsterdam, The Netherlands; 4 Department of Otolaryngology/Head and Neck Surgery, VU University Medical Center, P.O Box 7057, 1007 MB Amsterdam, The Netherlands

**Keywords:** Acceptance and commitment therapy, Self-compassion, Cancer, Oncology, Partner, Web-based interventions, User experiences, Qualitative, Interview

## Abstract

**Background:**

Partners of cancer patients are the cornerstone of supportive cancer care. They assume different roles and responsibilities that optimally support the patient. Such support is highly demanding, and many partners report (mental) health problems. However, many of them do not use professional supportive care themselves. Offering a Web-based self-help intervention based on Acceptance and Commitment Therapy (ACT) and self-compassion could be an important resource to support this group. This qualitative study aimed to examine user-experiences with a Web-based self-help intervention based on ACT and self-compassion among partners of cancer patients.

**Methods:**

Individual in-depth interviews, about partners’ appreciation of the intervention and lessons learned, were conducted with 14 partners of cancer patients who used the Web-based self-help intervention. Interviews were audio-recorded, transcribed verbatim and analyzed by three independent coders both deductively and inductively.

**Results:**

In general, partners appreciated the intervention, however, they also expressed ambivalent feelings towards peer support, the content of the feedback of their counselor, and the ‘tunneled’ structure of the intervention. The majority of the partners reported being more self-compassionate accepting that they experienced negative thoughts and feelings, they reported that they learned to increase the distance between their thoughts and themselves, they indicated being more aware of their personal values, and they thought that they were better able to commit to those values. They also reported other (non-specific) helpful processes such as insight and acknowledgement, positivity, the possibility to tell their story, time for themselves, and feeling closer and more connected with their partner (the patient).

**Conclusions:**

Partners of cancer patients indicated to appreciate the Web-based self-help intervention based on ACT and self-compassion. They felt that the intervention helped them to cope with negative emotions, thoughts, and one’s suffering; to practice self-kindness; and to clarify values based on difficult recent experiences. In addition, they felt that the intervention supported them to obtain insight and acknowledgement, positivity, to tell their story, make time for themselves, and feeling closer and more connected with the patient. We think that a Web-based psychological intervention based on ACT and self-compassion may be a valuable contribution in supporting partners of cancer patients.

## Background

Partners of cancer patients have to cope with multiple challenges, including the fear of an unpredictable future and possible death of the patient, feelings of guilt, inadequacy (always wishing to do more), self-doubt and frustration [1]. High levels of distress and caregivers’ strain have been demonstrated in many studies (e.g., [1–3]). However, offering support to partners can be challenging as most partners are extremely busy (e.g., [1]). Web-based interventions could be a solution as they are easily accessible and flexible to use [4]. Therefore, we developed – in close cooperation with partners of cancer patients – the Web-based self-help intervention *Hold on, for each other*. This intervention is based on Acceptance and Commitment Therapy (ACT; [5]) and self-compassion. ACT is a distinct model of behavioral therapy that aims to improve the psychological flexibility of people. It helps people accept what is out of their control (e.g., distressing feelings) and to commit to actions that enrich their lives [6]. The acronym ACT stands for (A) accept your thoughts and feelings, and be mindfully present; (C) choose values that are important in your life (e.g., openness); and (T) take action towards your values (e.g., sharing emotions openly with the partner). Another important component of ACT is cognitive defusion, which is not focused on changing the frequency or content of negative thoughts, but rather to change the relationship people have with their thoughts [7]. ACT has been proven effective for a variety of problems, including chronic pain [8], depression and anxiety [9–11]. However, as far as we know, it has not been applied in interventions for partners of cancer patients despite being potentially useful. Partners of cancer patients are often entangled with unhelpful thoughts (such as “What if the cancer comes back?”) and distressing feelings such as guilt, fear and anxiety (e.g., [1]). We think that ACT can help partners to experience these difficulties without resisting them, allowing them to live according to their values, applying them as corresponding actions in their lives and relationships, despite the barriers that can occur due to the cancer.

Self-compassion is defined as the ability to hold one’s feelings of suffering with a sense of warmth, connection and concern [12, 13]. Neff [12, 13] proposes three major components of self-compassion: self-kindness versus self-judgment, common humanity versus isolation, and mindfulness versus over-identification. According to Neff [12, 13], self-kindness refers to the ability to treat oneself with care in times of distress rather than harsh self-judgment. Common humanity entails recognizing that imperfection is a shared aspect of the human experience rather than feeling isolated by one’s failures. Mindfulness in the context of self-compassion is about holding one’s painful thoughts and feelings in balanced awareness rather than over-identifying with them. Research has demonstrated that higher levels of self-compassion are related to greater psychological health (in terms of less depression and anxiety) [14], greater happiness, optimism and life satisfaction [15, 16], greater relational well-being [17] and appears to be a powerful source of coping and resilience in times of distress (e.g., [18]). Although self-compassion has not been studied in the context of partners of cancer patients, it might particularly be useful for them because they often have unrealistically high expectations of themselves [1, 19, 20] and many also experience feelings of guilt for not doing enough for their ill partner or whenever they engage in personal activities even while their partner is suffering [1, 21]. Self-compassion may help partners of cancer patients renew their physical and emotional energies (e.g. through taking some time off from caregiving activities and spending some personal time), and enhance their emotional resources (e.g. resilience), both vital activities when coping with the challenges of being a caregiver [22].

ACT and self-compassion are closely related. Both approaches focus on improving mindfulness, helping people to defuse from their negative thoughts, and stimulating them to live in accordance with their personal values [23]. However, when compared to ACT, self-compassion is more explicitly focused on developing “the capacity for awareness of suffering and the motivation and ability to alleviate or prevent suffering we encounter” [23]. Considering the fact that partners of cancer patients often have high expectations of themselves and they would do anything – regardless the consequences for their own health – for their ill partner [24], self-compassion can particularly be relevant to them and the combination of ACT and self-compassion seems suitable for this target group.

Although ACT and self-compassion are increasingly being used as a theoretical framework for supportive (Web-based) interventions for a number of conditions (e.g., [8–11, 25]), most studies have been quantitative. Qualitative research is essential because it can reveal recommendations for improvement of interventions and valuable information about reasons for success and failure of an intervention [26]. Qualitative results can also provide insights into what the active ingredients of an intervention are [27] and if the underlying theory is appropriate [26].

The aim of this qualitative study was to explore the user-experiences with a Web-based self-help intervention *Hold on, for each other* among partners of cancer patients. The following questions were addressed: (1) Which elements of the intervention were appreciated by the participants and what suggestions do they have for improvements? (2) What did participants learn from the intervention? The results of this study will help to interpret the effectiveness of Web-based self-help interventions targeting partners of cancer patients (Table 1).

**Table 1:** Focus of the lessons, underlying theories and example exercises of *Hold on, for each other*

Lesson	Focus	Underlying theories	Example exercise
Coping with your emotions	Focus on emotional consequences of being a partner of a cancer patient. Partners learn how to recognize, allow and express their emotions.	Acceptance, Self-compassion/Mindfulness	*How often do I put on a brave face?* **Description**: We asked partners to write down emotional situations they have experienced, how they felt at that moment when the situation occurred and how they coped with it. **Aim**: To help partners to become aware of their own emotions and their coping mechanisms. Are they regularly putting on a brave face and suppressing their emotions?
Your resilience-plan – how can you keep going?	Focus on resilience. Partners learn how to manage a period of chronic stress and to improve their resilience.	Acceptance, Self-compassion/Mindfulness	*How much do you demand of yourself?* **Description**: We asked partners to write down how many hours they work, sleep, and have leisure time each week. **Aim**: To show partners how much they demand of themselves and if their planning is realistic.
My mind works overtime	Focus on worrying and negative thoughts. Partners learn how to cope with dysfunctional thoughts.	Cognitive defusion (changing relationship with thoughts), Self-compassion/Mindfulness	*Name your thoughts.* **Description**: We asked partners to write down the five most often occurring thoughts that are associated with the disease of their partner. Afterwards, they are asked to read the thoughts aloud and to pay attention for what he/she experiences. Then they are asked again to read the thoughts aloud but now with the addition “I have the thought/ feeling that…”. They are asked again what their experiences are. **Aim**: To help partners create a greater distance between their thoughts and themselves, and to show them that thoughts are not necessarily a reflection of reality.
What is now really important?	Focus on values in life and relationships. Partners learn about their personal values and how to live in accordance with those values.	Values, Self-compassion/Mindfulness	*Values in your relationship.* **Description**: We asked partners to write down those things in their relationship that they value the most. **Aim**: To make them aware of things that are not congruent with their values. Are there things that should be different? Is it worth investing in their relationship? What can they do to bring their actual life choices closer?
Afraid, tired and moments of joy	Focus on positive things in life and their relationship. Partners learn about how important moments of joy and positive emotions are in this difficult period in their lives.	Committed action, Self-compassion/Mindfulness	*Celebrate your relationship.* **Description**: We asked partners to choose activities (e.g., write a love letter, have dinner at their favourite restaurant). **Aim**: To make them aware of how precious their relationship is and how to live in accordance with their values.
The art of communication	Focus on communication. Partners learn how to improve their communication skills.	Communicating about what really matters, Self-compassion/Mindfulness	*What would you like to talk about?* **Description**: We asked partners to write down topics they have discussed lately with their partner, topics that have not yet been discussed, and - if so - why these topics have not yet been addressed. **Aim**: To stimulate partners to communicate about the things that really matter.
Optional Lessons			
Moving on with life	Focus on challenges that can occur after a successful cancer treatment. Partners learn how to cope with these upcoming challenges.	Acceptance, Cognitive defusion (gain control over thoughts), Values, Self-compassion/Mindfulness	*Increase your hope.* **Description**: We asked partners to imagine the situation in which their partner is cancer free for almost a year, and that he/she is feeling all right. They - as a partner - have done everything possible to cope with the situation. They have accepted it, and they are moving on with life. We ask them to imagine how life might be under these conditions. **Aim**: To show them that it sometimes can be helpful to create some distance and to have a closer look at their situation from a different point of view.
A good last period	Focus on topics related to the terminal phase of the patient. Partners learn what they can do in order to have a good last period with their ill partner.	Acceptance, Communicating about what really matters, Committed action, Self-compassion/Mindfulness	*Beautiful memories.* **Description**: We asked partners to think about (alone or with their partner) what they can do to produce new memories (e.g., things to experience together, trips or activities to make). **Aim**: To synthesise various aspects previously explored. To accept the development of the disease, talk about what really matters at that particular moment, commit to values and live in accordance with them.

## Methods

### Study sample and procedures

Partners of cancer patients were recruited from an ongoing randomized controlled trial (RCT) [28]. More detailed information about this RCT is presented in the study protocol [28]. At the moment of data collection for the current study, 52 partners of cancer patients had completed the *Hold on, for each other* intervention as well as the three- and six-months measurements after the baseline measurements. Of this group, 30 partners had indicated that they were willing to participate in the interview, and we randomly selected 20 partners for this study. Partners were contacted by e-mail and invited to participate in a telephone interview about their experiences with *Hold on, for each other*. Attached to the e-mail, they received the interview questions as well as a short summery of every lesson. With partners who were willing to participate, an appointment for a telephone interview was made. In total, 14 interviews were conducted. Five out of the twenty partners could not be reached and one partner withdrew from participation. The personal characteristics of the partners and the cancer-related characteristics of the patients are listed in Table 2. The mean age of the partners was 55 years old, and the majority were female, highly educated and employed. The patients were diagnosed with a variety of cancers. In most cases, the diagnosis was 1-5 years ago, and the partners mostly stated that their ill partner was unlikely to be cured. The time since participation varied among participants (mean time since intervention was about 8.6 months).

**Table 2:** Personal characteristics of the partners and cancer-related characteristics of the patients (*N* = 14)

Characteristics	*N*
Gender (female)	11
Age years, mean (S.D.); [range]	55.3 (9.3) [34-68]
Country of birth (the Netherlands)	14
Education	
Low	2
Middle	6
High	6
Employment	
Employed (>20 hours per week)	8
Unemployed/retired	6
Children	
No/or living away from home	10
Yes, living at home	4
Type of cancer	
Colon cancer	2
Kahler’s disease	2
Lung cancer	2
Prostate cancer	2
Leukemia	1
Bladder cancer	1
Lymph node cancer	1
Pancreatic cancer	1
Head- and neck cancer	1
Breast cancer	1
Time since diagnosis	
3-6 months	4
1-5 years	8
5-10 years	1
> 10 years	1
Treatment	
No	5
Yes	9
Stage of disease	
Patient is still in treatment with curative intent.	4
Treatment with curative intent is completed; patient is recovered.	1
Patient will (probably) not recover.	9

Once the partners had given their oral informed consent (written informed consent had already been given in the context of the RCT), the interview took place. The interviews were conducted in Dutch by a masters student of Health Psychology (JJ), who had been trained in conducting interviews. During the first three interviews, the student was supervised by a psychologist and the researcher of the RCT (NK). We decided not to include more participants when we found that no new information was found in the last three interviews, indicating that data saturation had been reached after the 14 interviews [29]. All interviews were audio-recorded and transcribed verbatim. The first author of this paper (NK) checked in a random sample of five interviews if the recordings were transcribed accurately. This could be confirmed. The interviews took between 15 and 60 minutes, with an average duration of 30 minutes. Testimonies appearing in this article have been translated from Dutch into English by an outsourced professional (native) translator. Personal characteristics of the partners and cancer-related characteristics of the patients were gathered in the context of the RCT.

### Description of Hold on, for each other

**Fig. 1:**
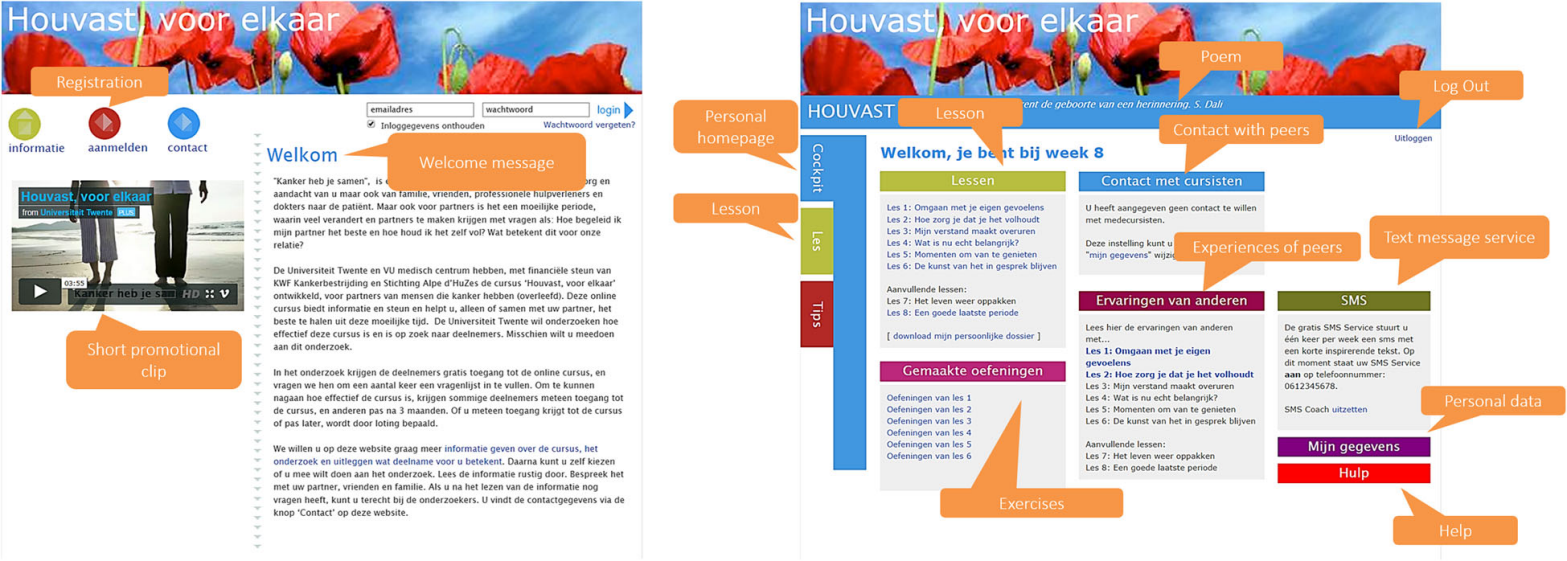
Screenshots of the *Hold on*, for each other website and personal homepage

*Hold on, for each other* (see Fig. 1 and Table 1) is an intervention that aims to help partners to positively persevere during the difficult times they find themselves facing. Built and co-created with partners in order to ensure it complies with their needs and wishes, the intervention consists of six lessons, and in each lesson one particular theme is discussed (see Table 1). The intervention makes use of tunneling, which means that partners were guided through the intervention [30]. First-time users were tunneled through two information pages in order to introduce them to the different components of the intervention. The content of each lesson and the lessons themselves were delivered in a predetermined sequence of steps. A next page or lesson could only be accessed when the previous page or lesson was completed. The aim of this tunneled structure was to enhance the change process of the participants by offering them the most appropriate intervention content at a particular moment in time [31].

Every lesson starts with a short text about the topic of that lesson. The core messages of ACT and self-compassion are integrated in these texts (see Table 1). The texts are enriched with short psychological exercises that are based on ACT and self-compassion. Each lesson also contains a mindfulness exercise based on self-compassion, that can be read or downloaded as a mp3-file. Partners also receive practical information, tips and references to relevant websites. Users can freely decide if they want to receive text messages (one per lesson) with short inspiring texts. Moreover, partners have different options to come into contact with peers. Previous studies pointed out that people often fear peer contact because they are afraid of being confronted with negative stories [32, 33]. To minimize this risk, *Hold on, for each other* offers three kinds of peer support. First, partners can share their answers on some exercises with other participants (and read the answers given by other participants). Second, they can share (and read) tips and advice, and third, they can contact other partners by sending a private message (message system is integrated in the website of the intervention). All the components are optional, and partners can decide freely to use them or not. Partners also receive support: automated support or personal support (weekly feedback messages from a personal online counselor (a trained masters psychology student of the University of Twente, The Netherlands), explained in more detail in study protocol [28]).

### Interview scheme

A semi-structured interview scheme was used. Partners of cancer patients were asked about experiences regarding the: (a) Web-based intervention in general; (b) psycho-education (lessons and psychological exercises); (c) mindfulness exercises; (d) peer support; and (e) practical information, tips and references. For each of these topics, partners were asked about what they appreciated, if they had any suggestions for improvements, and what they had learned. During the interview, partners were encouraged to motivate their answers and experiences.

### Data analysis

Two coders (JJ, NK) analyzed the data with the use of open coding, axial coding and selective coding, applying deductive and inductive analysis [34, 35]. First, the coders independently selected relevant fragments and coded them in one of two predefined categories: evaluation of the intervention and the lessons learned of the intervention. Afterwards, inductive analysis – meaning that subthemes derived from data, instead of predefined categories – was further used to categorize all fragments into subthemes. After every five interviews, the coders met to discuss their subthemes. When dissimilarities were found, the two coders reached a consensus and the coding scheme was redefined. After all codes have been obtained, code schemes with exemplary codes were developed by constant comparison of similarities and differences in the data [35]. When the two coders finished their analysis, a third researcher (CHCD) checked the categorization and the three researchers further discussed any disagreements about the categories. Final categories were defined on the basis of consensus between the three researchers.

## Results

### Partners’ evaluation of the intervention

In the next sections, the partners’ evaluation of the intervention is described, starting with more general comments and followed by their evaluation of the following components of the intervention: psycho-education (lessons and psychological exercises); mindfulness exercises; peer support; practical information, tips, references and text message service; and personal support (see Table 3). Some partners mentioned arguments for both why they appreciated a specific element or why they did not appreciate it. Therefore, the number of participants not always adds up to 14. In addition to the evaluation, an overview of the partners’ suggestions regarding both the improvement of the intervention and a dissemination of such an intervention is provided.

**Table 3:** Partners’ evaluation of specific parts of the intervention

Category	n^a^ pro	Pro argument	n^a^ against	Against argument
Psycho-education	14		10	
		- General comments; unspecified		- Not personally relevant
				- Particular exercise not appealing
Mindfulness exercises	10		3	
		- Exercises are pleasant, fun, interesting and relaxing		- No need for mindfulness exercises
				- Mindfulness exercises are not appealing
Peer support	3		12	
		- Possibility to exchange tips and experiences with peers		- No need for peer support
				- Sufficient support from personal network
				- Afraid of being confronted with negative stories
				- No capacity to listen to the story of another partner
				- Doubting that peers could help each other
				- Preference to stay anonymous
				- Web-based support felt too impersonal
				- Afraid that personal situation is not comparable to that of others
Practical information, tips and references; text message service	6		3	
		- Pleasant to read		- Information not personally relevant
		- Useful information		- No need for this information
				- No time to read long texts
Personal support	1^b^		4^b^	
		General argument; not further specified		- Preference for more personal feedback instead of feedback on progress using the intervention
				- Preference to have the opportunity to talk to a professional from time to time
				- Language of feedback messages was not appealing

^a^Participants could give reasons for both why they appreciated a specific component or why they didn’t appreciate it. Therefore, the numbers not always add up to 14

^b^Six of the partners received support from a personal counselor during the intervention. Five of them talked about what they appreciated or didn’t appreciate about this element

The general comments were divided into six categories: (1) unspecified; (2) structure/design; (3) topics addressed; (4) flexibility; (5) anonymity; (6) (positive) approach. Unspecified comments included quotes such as *“I thought it was a very interesting course.”* Seven partners mentioned that they appreciated the structure and design of the intervention: as illustrated by the following comment from a partner” *“I am always amazed how well it is made, and how many great elements it comprises.”* However, four partners were less satisfied with the structure and the fact that they were guided through the intervention, because they felt it was unclear or did not fit their needs. As one of the partners said:

*I noticed during the course that it was difficult to adapt my life and its rhythm to the rhythm of the course. Of course one does not have exactly those needs in exactly that order… I can imagine it was carefully thought out, but it did sometimes feel like someone was stepping on the brake.*

Five partners particularly appreciated the topics addressed in the intervention, because they fit their personal situation and were highly relevant for partners of cancer patients. One partner mentioned: *“The words ‘Hold on’ [in the intervention’s name] say it all, since it is something to ‘hold on’ to. Wherever or whenever, people profit from it. For one person, this element is important, for another person, another element.”* Four partners indicated that certain topics were not that relevant for them, as one partner mentioned in the following quote: *“Some parts made me think, this doesn’t mean anything to me.”*

Four partners liked the flexibility of the intervention. They appreciated that no exercise was mandatory, but that you could freely decide what you wanted to use and how you wanted to use it. One partner said: *“I appreciated being able to follow at my own pace, to be busy with … let’s say … formulating my thoughts and feelings.”*

Two partners liked that they could stay anonymous, and one partner particularly liked the positive nature of the intervention and the way partners were approached:

*Also the care with which [the researchers designed and formulated] everything. Yes, I think it deserves a big A. Also how you [designed the intervention to] approach people, in such a pleasant way. In any case, the threshold is low, so one does not get the feeling one is doing things the wrong way.*

#### Evaluation of psycho-education (lessons and psychological exercises)

While the partners did not cite any particular lesson, they all mentioned reasons why they liked the psycho-education. Five partners reported an appreciation for general aspects. For example: *“And then I sent it off and waited excitedly for the next session”, or “We participated with a lot of pleasure and greatly profited from it.”* Other partners highlighted why they liked a particular topic or exercise. For example, one partner appreciated *“… the first lesson, in particular, especially because it made you express your feelings and put them into words, since we are always so busy with other people.”* Another partner said: *“And that lesson* [6] *arrived just in time for me, which struck me again. Just spit it out and type or write it down, and then you are rid of it…”*

Ten partners also mentioned some particular aspects of the intervention that they appreciated less. Four partners mentioned that (some parts of) the psycho-education were not relevant for them, as illustrated in the following quote: *“I can’t name any examples now, but there were questions that I kind of skipped, though I don’t remember what they were. I didn’t think they applied to me.”* Five partners indicated that they did not like one particular exercise, called “The answers to what-if questions,” for numerous reasons. One felt that this exercise was particularly unappealing, for example: *“I always consider what-if questions senseless.”* Another felt the exercise was too negative: *“I’m not busy with such what-if questions, but try to think in terms of solutions. So I don’t always worry like ‘imagine that …’ I’d rather think in terms of positive solutions.”* Still others felt that such what-if questions were too difficult to think about. One partner explained: *“I really found what-if questions quite complicated.”* However, this exercise also helped some partners to cope with difficult thoughts, which we later describe in the section about long-term effects.

#### Evaluation of mindfulness exercises

Ten partners appreciated the mindfulness exercises because they experienced them as pleasant, fun, interesting and relaxing. One partner explained this appreciation as follows: *“Yes, very pleasant. In the beginning, at first, I thought: what I am doing here? But then gradually… it was very pleasant.”*

Three partners were more critical, indicating that they had no need for such exercises or that the exercises were not appealing to them, for example: *“I didn’t have much use for it. At that moment, I was too busy for it, and anyway, it wasn’t really my thing.”*

#### Evaluation of peer support

Two partners appreciated the possibility of having contact with peers because they were interested in sharing their tips and experiences and reading the tips of others. As one of them said: *“They offered tips, and I always read those, which I thought was really great.”* However, the majority of the partners did not use the peer support, and some of the partners had multiple reasons for not doing so. Reasons for this included that six partners did not need peer support or three of them mentioned that they had sufficient support from their private network, as one partner explained: *“I didn’t feel the need for it, since I am in such close contact with my friends and family.”* Other reasons for not using the peer support were: three partners were afraid of being confronted with negative stories; three partners had no emotional energy for the story of another partner; two partners were doubting that peers could help each other; one partner wanted to stay anonymous; for two partners peer support via the intervention felt too impersonal; and two partners were afraid that their situation was not comparable to that of others, as is illustrated in the following quote:

*It was also difficult for me, because I realized that amongst the fellow participants, there were some whose partners were terminally ill. Since that is not my situation, I feel very privileged and would not dare to come forward with my things, which are really not that bad… You could also read about the situation they were in, which, yes, made me feel ashamed of sharing my things.*

#### Evaluation of practical information, tips and references, and text message service

Six partners appreciated the practical information because it was pleasant to read and contained useful information as illustrated in the following comment of one of the partners: *“I recall those tips… those summaries of all the information. I did appreciate all those practical tips a lot, just reading through them once.”* Two partners mentioned that they appreciated the text message service. One of them described the appreciation as follows: *“I really liked them and also showed them to [my partner], like: ‘Now see what I got!’”*

Three partners did not like the practical information, because one of them felt that the information provided was not relevant for them, another one was not in need of such information, and one partner had no energy to read long texts: *“I was not in the mood to read long texts, so I just forgot about it.”*

#### Evaluation of personal support

Six of the partners received support from a personal counselor during the intervention. One participant didn’t mention if he/she appreciated this component. One participant reported being satisfied with the personal counselling: *“Yes, that was very nice, it was really nice to get personal support.”*

Four partners were not entirely satisfied with the support provided by their counselor because they had expected personal feedback instead of feedback that mostly targeted their progress in the intervention. They would have liked to have the possibility to talk to a professional from time to time, for example via telephone, because then they might have the opportunity of discussing matters more deeply. This contact could also function as a motivator to stay engaged with the intervention. One partner felt that the language of the feedback messages was obviously language social workers use:

*‘How good of you’ and ‘Nice to hear’ and ‘Oh well, that doesn’t matter.’ Yeah, I thought, come on, I’m 60. A further disadvantage is that I am a social worker myself, so that is really social worker’s language that really doesn’t work for me.*

#### Suggestions for improvement and dissemination of the intervention

In addition to the suggestions for improvement of the intervention (less rigid structure of lessons and more personal support), one partner also indicated an interest in a book version of the intervention. In addition, three partners mentioned that they would like to see an additional lesson about bereavement, as illustrated by one of the partners: *“From my personal experience, I would perhaps have liked an extra lesson that went more into detail about how things are once your partner has passed away. I mean, like an optional lesson.”*

We also asked partners to reflect on how we could improve the dissemination of such an intervention. Twelve partners said that they would have preferred an introduction of the intervention via a healthcare professional (such as an oncologist, nurse or social worker) in the hospital. There were different preferences regarding the optimal moment for introducing the intervention. Based on the answers provided by the partners, it appeared that every partner has his or her own moment in the cancer trajectory when support is needed, indicating that it might be important to offer the partners such an intervention at various times. This need is illustrated by the following testimony:

*When somebody hears that [the cancer] has spread and there is nothing that can be done about it – I would not say something like, “Well, we offer a course you might like to attend.” In any case, I would wait a little while and then, during the next stage [of the disease], offer the course a few times or at least point it out: “This is there for you, but if you don’t want to make use of it, that’s fine. But this is what we do offer.” For indeed, there is so much attention on the patient and really no attention for those nearest [to him].*

### Lessons learned from the intervention

In response to the question regarding what the partners learned from the intervention, eleven partners answered in general terms such as: *“I greatly profited from it. Not all parts were successful, not all lessons were effective, but still I greatly profited from it.”* Seven partners indicated that the intervention encouraged them to think about their situation. Four partners noted that the intervention was offered at a moment when they particularly needed help, and they liked the fact that they finally had received some attention, for instance: *“I was so happy this came onto my path for, as a partner, I was so sad and worried. Of course, all the attention goes to the sick partner and only very few people really ask, ‘And how are you doing?’”*

Specific lessons learned could be divided into two categories: (1) lessons learned on the short-term that are processes that partners had experienced during or directly after the intervention and (2) lessons learned on the long-term or experiences regarding their well-being and relationship that partners were still experiencing at the time of the interview.

#### Lessons learned on the short-term

Lessons learned on the short-term could be divided into five categories: (1) insight and acknowledgement, (2) ACT- and self-compassion-related, (3) guidance and solutions, (4) positivity, and (5) attention as the caregiving partner (see Table 4).

**Table 4:** Lessons learned on the short-term

Short-term effects	Total n	Subthemes	Subtheme Total n	Example quote
Insight and acknowledgement	13	Better understanding of the situation (psycho-education).	10	“There were many emotions that I recognized, as you know, but reading about them made me think, ‘Yes, that’s how it is.’ Like the expression ‘emotional roller coaster,’ which suddenly appeared to be true also for me. One is familiar with the phrase, but then suddenly it becomes part of one’s life.”
		Experiencing acknowledgement and recognition.	8	“I also felt confirmed in my feelings. So I am not crazy, this is normal, this is a phase in my life and a process to go through and that all people in my situation have to face.”
		Confirmation of their ability to cope with a difficult situation.	4	“I felt like I had done really well. By following the course, I discovered that this feeling of mine had been quite right, for things did indeed go well.”
ACT- and self-compassion-related	11	Be more (self-) compassionate.	7	“So indeed, if I remember what was said, you know like ‘Do not demand too much from yourself,’ ‘Take time to relax,’ and the other sources of help, then I think: ‘Yes, that is right.’ It really helped me a lot.”
		Be more mindful.	7	“It was really good to sometimes be really quiet, just to listen for a moment and feel my feelings and be quietly present to myself for some time.”
		Creating distance from your own thoughts.	5	“It was like letting your thought-factory go on a holiday, so to say. I put [the mindfulness exercise] on my mp3 player and sometimes took it with me to bed. So when I could not sleep, I would play it.”
Guidance and solutions	9	Guidance to improve communication.	8	“They were very difficult subjects about which we first explored independently from one another. We then talked with each other about our answers.”
		Helpful solutions.	3	“Those exercises helped me to experience my meetings with friends as relaxing. And indeed, why wouldn’t you share your worries?”
Positivity	6	Positivity.	6	“Well, we consciously looked at what we had done together, also at the positive things, even though it was not all that positive. Still, we said: ‘We haven’t done so badly, you know?’ Small things, they don’t need to be so big.”
Attention for me as a partner	5	Moments for myself.	3	“Those are the moments you hold on to, you know? When you think: ‘Yes, how wonderful! I feel so relaxed now.’ Those are the moments, during the short time one has, that you try to make your own.”
		Telling my story.	3	“The course really helped, because I was able to talk and write about it and thus gain distance from it all. In daily life one meets so very few people with whom you can share your story. And in this case, I was able to share it.”
		Structure.	1	“It provides one with so many handles. And also with some structure in all the chaos, so to say.”

##### Insight and acknowledgement

Ten partners reported that the intervention helped them to better understand their situation and four of them found confirmation of their ability to cope with the difficult situation.

Eight partners indicated that it was pleasant to be acknowledged as a partner and to realize that their feelings and emotions are “normal” and not uncommon in their situation, for example:

*The realization that one understands what is happening inside, by asking ‘Why do I behave in this way now?’ and ‘Can I behave in a different way?’ worked as a self-reflection. It was self-reflection, but also helped others [other people in this partner’s life] to understand that it [this partner’s behavior] is not unusual …*

##### ACT- and self-compassion-related

Half of the partners reported having a positive experience with the mindfulness exercises. These exercises gave them rest, peace, time to reflect, and time to be fully aware of themselves and their surroundings, as illustrated in the following quote of one of the partners: *“Indeed a short moment for oneself. Just for a moment, let me call it a conscious moment, or how shall I call it? A moment of recollection, one in which you really get quiet.”*

Seven partners indicated that they learned to be more (self-)compassionate. The intervention taught them to be more kind and caring towards themselves, to ask for help and to share their experiences with others. One partner said: *“I told myself: ‘That’s not proper. You shouldn’t enjoy yourself, because you’ve been through something very unpleasant.’ And then I heard during the lesson: ‘On the contrary, you really should enjoy yourself.’ And that helped me a lot.”*

Five partners reported that the intervention helped them to create more distance from their own thoughts, as illustrated in the following quote:

*Looking at oneself and, in some way, taking distance from oneself. If I may speak about Lesson 7, at a certain moment it says: ‘If you were ten years older now, and you were still the same person with the same experiences, how would you look at yourself and which tips would you give yourself?’ That was such a powerful way to take some distance from oneself: now I am ten years older and I am going to give myself some tips, which you can only formulate if you can take a distance. That is such a freeing experience, such a … I can’t explain it, but it’s so incredibly good!*

##### Guidance and solutions

Most of the partners mentioned that the intervention provided guidance and useful solutions. In particular, the guidance to improve their communication with the patient was mentioned by eight partners. The texts and exercises stimulated conversations and provided an effective method to talk about sensitive topics like negative emotions and positive experiences, such as recollecting memories of special events like holidays or weddings. To illustrate this sub-category, one participant reported:

*We talked about things we had not mentioned for a long time. It’s like you protect each other. You don’t tell each other certain things, because the situation is so difficult and you’re just surviving. Things that are really important, things that were important before – you don’t think of them any longer. The lesson pointed this out to us, so we started talking about it all and that was so incredibly good.*

Three partners mentioned that the intervention also provided helpful solutions for problems they had. Two indicated that the intervention provided useful tips on how to ask for help from the social network or how to decline it. Another partner indicated that the intervention provided some guidelines on how to become more self-reflective and that this self-reflection could help adjust one’s behavior.

##### Positivity

Six partners indicated that the intervention helped them to think and act more positively. One partner mentioned that it was fun to do the intervention because it brought new insights and was enjoyable. Another partner mentioned that the intervention brought positivity, but did not specify further. Three partners liked the fact that through the intervention they felt more connected with their partner. They were more aware of the positive aspects in their relationship and they valued these even more. Another partner reported having become more aware of the little things in life that can make a difference:

*I followed that lesson during a particularly difficult time, and it greatly helped me to carefully think about the whole situation. Also about the rays of light, for it all seems so hard. And when I thought about those rays of light, I told myself: “Oh yes, remember? There still are so many nice things!*

##### Attention for me as the caregiving partner

Three partners reported that the intervention gave them the opportunity to have some time for themselves, and three partners valued the possibility to tell their story, as illustrated in the following quote: “*There just happened to be elements in the course that simply were of great importance to me, especially the opportunity to tell my story.”* One partner felt that the intervention offered some structure during what felt like a chaotic and difficult time.

#### Lessons learned on the long-term (experiences regarding well-being and relationship)

Lessons learned on the long-term are experiences that the partners were still experiencing at the time of the interview. These can be divided into four categories (listed from the most to least mentioned): (1) ACT- and self- compassion-related skills, (2) positivity, (3) connectedness, and (4) personal growth. The categories and their subcategories are listed in Table 5.

**Table 5:** Lessons learned on the long- term (experiences regarding well-being and relationship)

Long-term effects	Total n	Subtheme	Subtheme Total n	Example quote
ACT- and self-compassion-related	14	More (self-) compassionate.	11	“What is it all about? At the side of a sick partner, it is also about oneself. Yes, I see this also as a parent, you know. It is a combination of things, also in your role as an employee. As a partner too, but all of that can only happen if you stay in it also for yourself.”
		More aware of the here and now/more relaxed.	8	“Just looking at things, not specifically, but with more awareness, so to say.”
		Changing relationship with thoughts (cognitive defusion).	8	“Yes, some relaxation. At night you lie awake, and you try to remember the course and then bring some quiet to your thoughts and feelings.”
		Living in accordance with personal values.	7	“Yes, just say: ‘I don’t feel like it’ or ‘I don’t have time for that’ or ‘I won’t make time for that.’ “
		More acceptance of emotions.	6	“Through the lessons I somewhat learned to think: Yes, it is indeed very difficult and I is alright if I feel sad sometimes.”
		Better communication about what really matters.	4	“It is also good to talk about it with my husband. He doesn’t talk very easily, but thanks to the topics that were included, we also learned to really talk to each other.”
		Awareness of values in life and relationship.	4	“Especially ‘what is really important.’ One tends to easily to just continue in the same old way.”
Positivity	9	Positivity (enjoy the little things).		“It is still a source of inspiration for me, just to think about positive things and really dwell on them, like enjoying the sunshine while taking a walk and those kinds of things. Simply with real awareness.”
Connectedness	6	Spouses got closer (more connected).		“In any case it brought us together for a conversation. From both sides, so to say, with the right instruments to better understand each other and to help each other during the whole process.”
Personal growth	3	Stronger and more resilient.		“Through the course, you move forward a bit and you grow. And the essence – you know it and you keep it alive.”

##### ACT- and self-compassion-related skills

Eleven partners felt more self-compassionate after the intervention. They realized that it is important to also focus on oneself, to be kind and caring not only towards the patient, but also towards oneself, as is illustrated by this quote:

*I have also become a lot kinder to myself, also thanks to these lessons. Because I had to write down things and answer questions, I came to realize that I was also judging myself. And well, that is something I have done already for a long time. But now I naturally enter into a different phase of my life, and then it is so important to be very kind to myself, to allow myself some time to just do nothing for a little while. Or just to really take care of myself and pamper myself a little.*

Eight partners reported being more aware of their surroundings, and they felt more calm and relaxed after the mindfulness exercises. For example, one of them said: *“Also afterwards, the mindfulness exercise helped me to stay much calmer in everyday life… It helped me to be more conscious and calmer.”*

Eight partners reported that the intervention helped them to change their relationship with their thoughts, allowing them to see their thoughts just as thoughts and not the truth. One partner described this change:

*One of the tips one receives is to think with every thought: ‘That is a thought’ or something like that, for instance ‘I have thoughts that say…’ That is really good because it shows that a thought is not the truth. You think it and it is just an image. And that gives one some kind of strength. I feel stronger by those thoughts, by formulating them in this way.*

In addition, the intervention helped half of the partners to live in accordance with their personal values. Five partners indicated that they had learned to accept their needs, such as spending more time with their ill partner. Two partners indicated being stricter about protecting their personal boundaries. As one of them said:

*I learned that it is always easy to drop everything and be there for everyone else while forgetting oneself. It is not right that everyone else runs your life. You should be able to say: “Wait there for a moment and don’t come any further.*

Six partners reported feeling more acceptance towards their own emotions − like fear or worries, for example:

*I learned something from the what-if questions. At the time, I was very busy with these kinds of questions. During that lesson, I came to understand that one has to learn how to deal with them. What if this, what if that – you have to learn to give such questions their place. That doesn’t mean finding an answer to what-if, but rather that it is normal to have what-if questions and to know how to deal with them. That doesn’t mean they direct your life, but that you give them their place, like: “This is the question to which I, for the moment, do not have an answer and that’s that.”*

Four partners felt that they could better communicate about what really matters. The lessons stimulated conversations between partners and patients, and they felt that they remained more open towards each other, as is illustrated in the following quote:

*Yes, afterwards we were more open towards each other about topics we would not have talked about so easily before. After the conversation, we carried on in a different way, so it absolutely brought us something.*

Four partners reported that the intervention helped them to be more aware of their personal values in life and their relationship, For example:

*I came to understand that the most important thing is my family. That is what it is all about. I realized my values … I realized more clearly which are the most important values in my relationship and in my family situation.*

##### Positivity

Nine partners mentioned that they were more positive about their lives since they had completed the intervention. They were more aware of the small positive things in life that make life worth living. One partner said: “*And now I do see [those rays of light], yes! Perhaps I did so before without realizing it, it happens so spontaneously. And now I think: … Those rays of light make it all more pleasant.”*

##### Connectedness

Six partners mentioned that they became closer and felt more connected to their partner. They reported that the intervention helped them to reflect on their relationship. For example, they indicated to be more aware of the importance of talking openly about feelings, needs and wishes and not just assuming what the other person might think. One partner also mentioned that she was more aware of the affection she felt for her partner. Kissing him was not just an automatic routine anymore, but she felt that this kiss actually meant something to her and to her partner. The following quote is an example of the connectedness the partners mentioned in our study: *“It gave us a sense of belonging, like: Hey, we are actually quite happy together.”*

##### Personal growth

Three partners reported that they became stronger and more resilient as a result of the intervention. They mentioned that the intervention itself was a source of energy or it provided them with information about where they can find helpful resources in order to improve their resilience and personal strength. One of the partners said: *“Yes, during that time I really was … sad. It was all so difficult, but thanks to the lessons, I came to see that I am much stronger than I thought I was.”*

## Discussion

This study aimed to investigate the user-experiences with a Web-based self-help intervention based on ACT and self-compassion. We found that, in general, the intervention was positively appreciated and partners’ learned lessons appeared to be helpful in supporting them to cope with the challenging situations they faced. Our findings can partly be explained by processes related to ACT- and self-compassion that were the basis of the intervention.

Most partners reported lessons learned on the short- and long-term related to these theories. Partners, for example, mentioned that the intervention made them aware of the importance of not only focusing on (the needs of) the patient and being kind and caring towards him or her, but to also treat themselves with the same amount of compassion. Some partners also mentioned that the intervention helped them to be more mindfully present and to accept their (negative) emotions instead of over-identifying or avoiding them, which refers to the A(cceptance) part of the ACT-model. The acceptance of negative emotions and sensations has often been found to be related to better mental health (e.g., [36]). In addition, the intervention helped partners to improve their ability to have more control over difficult thoughts, by creating a greater distance between their thoughts and themselves. This process, called cognitive defusion, is one of the core processes of the ACT model and it seems to be particularly important for partners of cancer patients because they often over-identify with unhelpful thoughts and feelings [1]. This result is in line with previous studies among other populations [37, 38]. Bacon et al. [37] examined the active processes of ACT in people experiencing psychotic disorders, and Mathias et al. [38] focused on the ACT processes in chronic pain patients. Both studies found that cognitive defusion is an ACT process that facilitated change in their participants. Partners also mentioned that our intervention helped them to become more aware of their personal values, more effective in communicating their values with the patient, and better able to commit to these values. These findings refer to the “C(hoose)” and “T(ake action)” of the ACT model and show that values and committed action may help partners of cancer patients. It seems that these processes can help them reorganize and rediscover their personal values and to live in accordance with them, despite the barriers caused by the cancer. Previous research has identified these ACT processes as contributing to positive change in other study populations, such as people with psychotic disorders [37] and patients with chronic pain [38].

Besides the processes related to ACT and self-compassion, our intervention seems to have also helped partners via a number of other processes such as insight and acknowledgement, positivity, possibility to tell their story, time for themselves, and feeling closer and more connected with the patient. Although these factors are not specifically related to ACT and self-compassion, they may be an important consequence of acceptance and self-compassion. In this respect, the impact of positivity is perhaps the most interesting. Nearly two-thirds of the partners reported that the intervention helped them to become more positive in life: allowing them to become aware of the little things in their lives and relationships that make life worth living (e.g., enjoying the weather; going for a walk; quality time with the family; a good conversation). Gaining acceptance towards one’s emotions, becoming kinder towards oneself and acquiring awareness of one’s values may result in an overall broader awareness, an improved experience of positive emotions, and an increase in enjoyment and appreciation of the positive things that remain in one’s life. According to Fredrickson’s broaden-and-build theory [39, 40], recurrent experiences of positive emotions (e.g., gratitude, love, feelings of joy) can increase a variety of personal resources such purpose in life, self-acceptance, mindfulness and positive relationships with others [41]. These resources can consequently lead to an increased life satisfaction and decreased levels of depressive symptoms. This aspect seems particularly crucial for partners of cancer patients because they are often confronted with many negative emotions, uncertain future perspectives and a high burden of responsibilities [1].

Another interesting factor that our findings revealed is the possibility of telling one’s personal story. This factor seemed important for the partners because, by telling their story, they felt acknowledged. They also valued finally receiving some attention. During the patient’s illness trajectory as well as within the social network of the partnership, the main focus often lies on the patient. As a consequence, the needs and concerns of the partners are often overlooked, and they have a minimal opportunity to tell their story. Web-based interventions aimed at this group can offer the attention and comfort they are vitally missing.

Besides offering clues about processes of (positive) change, our study also provided valuable information about the partners’ appreciation of the intervention in general as well as specific elements. This information might be of interest for developers of future Web-based interventions for identical or similar groups. First, partners did not appreciate peer support as much as the other elements of the intervention. For example, they mentioned that they were not in need of peer contact or they were afraid of being confronted with negative stories. This result is in line with the outcomes of previous studies, which pointed out that people often fear peer support because they are afraid of hearing negative stories [32, 33]. While we tried to minimize this risk by offering different options of peer support, according to the users’ experience, it seems that this approach was not sufficient to reassure the partners. Second, whereas previous research showed that personal feedback about a participant’s progress is a valuable addition to an intervention [9, 42], the partners in our study mentioned that they would have liked the feedback to be more personal and to discuss some matters more deeply. For this specific group, more personal feedback could be important because, within the patient’s care environment, often little or no room exists for the partners to tell their story or to express their concerns and questions. A more blended approach of the intervention (a combination of Web-based and face-to-face components) could be a possible solution to this problem [43]. Third, whereas some partners particularly appreciated the ‘tunneled’ structure of the current intervention, others preferred a less strict structure. As described earlier, we chose to deliver the content in a step-by-step format with a predefined order because we thought that this particular sequence of modules would be most beneficial for the partners, as it would give the partners sufficient time to process all the information.

Finally, regarding the dissemination of this intervention, partners suggested that healthcare professionals in hospitals (e.g., oncologists, nurses or social workers) should introduce the intervention not just once, but several times during the patient’s cancer trajectory. Partners seem to have different needs regarding when they might want to obtain support. Some partners would like to receive a psychological intervention immediately after the diagnosis, whereas others want to participate when they think it is necessary. We have to consider that partners often do not ask for help. Often they are not aware of their own health problems or they even neglect them because the patient’s well-being is their utmost priority (e.g., [44–46]). We expect that by offering an intervention (at various stages) to the partners, they may be more aware of the fact that they are at risk of developing physical or psychological health problems and that receiving help is not unusual. This multiple introduction of the intervention may help to lower partners’ threshold of asking for help for themselves.

The results from our qualitative study suggest that the training of ACT-related processes and self-compassion can indeed help partners of cancer patients to cope with the challenging situation they are facing. The outcomes of this study need to be interpreted with caution. First, it could be possible that the partners who were willing to participate in this study were initially more positive or negative about the intervention than partners who did not want to participate. This might indicate that our results may not be generalizable. Second, results may not be representative for all participants of the intervention because of the rather small study sample. However, the personal- and cancer-related characteristics of this sample are comparable to the characteristics of all participating partners and we reached data saturation which may suggest that the results are reliable. Third, this study was retrospective, indicating that the results relied on participants’ memories of the intervention. As aforementioned, the time since participation varied among participants (mean time since intervention was about eight months). In anticipation of this possible limitation, before interviewing the participants, we provided all of them with a short summary of every lesson of the intervention. Yet, half of the partners still had difficulties remembering specific elements of the intervention, for example a specific lesson or exercise. However, all of the partners were able to describe general experiences with the intervention. A fourth limitation is related to the positioning and reflexivity of the researchers, since five of the researchers had worked with the randomized controlled trial. To minimize this bias, we included a sixth co-author (JJ) who was not part of the regular research team. JJ conducted and transcribed the interviews and was also involved in the data-analysis. Furthermore, we constantly reflected on our process when we coded and interpreted our data. We were cautious to not only present opinions of participants who were positive about the intervention, but to also display critical sounds regarding the intervention. Quantitative effect studies are, of course, necessary to evaluate the effects of the intervention on psychological distress, caregiver burden, mental well-being and other outcome measures. Therefore, we are currently conducting a randomized controlled trial.

## Conclusions

The Web-based self-help intervention, based on ACT and self-compassion, was appreciated by partners of cancer patients and helped them to cope with negative emotions, thoughts, and their own suffering; to practice self-kindness; and to clarify values based on their difficult recent experiences. In addition, the intervention supported them to obtain insight and acknowledgement, positivity, to tell their story, make time for themselves, and feel closer and more connected with the patient.
